# Job Quality and Work—Life Balance of Teleworkers

**DOI:** 10.3390/ijerph18063239

**Published:** 2021-03-21

**Authors:** Paula Rodríguez-Modroño, Purificación López-Igual

**Affiliations:** Department of Economics, Quantitative Methods and Economic History, Pablo de Olavide University, 41002 Seville, Spain; mplopigu@upo.es

**Keywords:** telework, remote work, mobile work, job quality, working time quality, work–life balance, work intensity, home-based work

## Abstract

As telework and mobile work arrangements become more widespread with new advancements in digitalization, these flexible models of work are rapidly expanding to new categories of employees and completely modifying working conditions and job quality. The aim of this study was to assess how particular types of telework affect different dimensions of job quality. We applied multivariable techniques to a sample of 35,765 workers from the Sixth European Working Conditions Survey. Our findings show that gender and types of telework by workplace and ICT-use intensity are crucial factors affecting working conditions and job quality. Occasional teleworkers are the group with the best job quality, while highly mobile teleworkers are those with the worst job quality and work–life balance. Home-based teleworkers, especially women, present better results than highly mobile workers in terms of working time quality and intensity, though in exchange for lower skills and discretion, income, and career prospects. This study contributes to deepening our knowledge on the impacts of flexible arrangements of work, providing an analysis of current data on different dimensions of job quality and work–life balance and including gender as a crucial axis of analysis.

## 1. Introduction

A growing number of tasks can be performed and surveilled anywhere and anytime with the help of new mobile information and communication technologies (ICTs) [1,2,3,4]. As labor markets transition to more flexible models of work with digitalization, working conditions are being completely altered [5]. Flexible telework arrangements affect working conditions, the work–life balance, performance, and prospects of workers in different ways [6]. On one side, telework offers workers more autonomy and flexibility, which usually leads to better work–life balance. Advocates of telework note its benefits in promoting female labor force participation, given women’s high unpaid care workloads [7,8,9,10,11]. On the other side, there can be disadvantages: It can lead to an intensification of work, longer working hours, and the overlapping of work and home life, which may be particularly harmful for women. This is the so-called “autonomy paradox” of such arrangements. 

Thus, studies on the association between flexible working practices, job quality, and work–life balance are still scarce and inconclusive [12]. One possible explanation for these contradictory findings is that existing studies do not usually distinguish between different groups of teleworkers, neglecting that the consequences of telework may greatly differ depending on the kind of remote location [13,14,15,16,17,18,19]. Hence, the aim of this article is to contribute to this debate, exploring the consequences that working remotely or telework has for several dimensions of job quality and work–life balance, focusing on two crucial axes of analysis: First, the differences by type of remote work, depending on place and frequency of flexible arrangements, and second, gender differences. Following Eurofound and ILO, we combined work location, level of mobility, and ICT use to categorize three main types of teleworkers according to their telework arrangements [6,15,16]: Regular home-based teleworkers, highly mobile teleworkers, and occasional teleworkers.

A second reason for the autonomy paradox may be due to an ambiguous definition of autonomy [14]. To avoid that, we examined the differences for our subgroups of teleworkers through quantitative multivariate analysis of five dimensions of job quality indices from the Sixth European Working Conditions Survey: Work intensity, working time quality, skills and discretion, prospects, and earnings. These job quality indices capture the multidimensional nature of the concept of job quality and reflect the fact that each dimension has an independent influence (whether positive or negative) on the health and wellbeing of workers [20]. 

In summary, this study contributes to deepening our knowledge on the impacts of flexible arrangements of work, providing an analysis of current data on different dimensions of job quality and work–life balance, and including gender as a crucial axis of analysis.

## 2. Literature Review and Hypotheses

Literature and empirical studies on the effects of flexible arrangements, such as different types of teleworking, suggest that these may have paradoxical consequences for workers’ work–life balance, job satisfaction, and wellbeing. On one hand, the use of ICT has led to huge gains in flexibility and agility and provided opportunities for greater autonomy, since teleworkers have supposedly greater flexibility on where, when, and how to work. According to social exchange theory, the more the job autonomy that teleworkers have, the greater the effort they put into their work [21,22]. Thus, employers gain from a more productive workforce that uses less space and is more cost-effective, and workers gain from the prospect of a better work–life balance, thereby increasing levels of job satisfaction and organizational commitment. Under this approach, telework arrangements are claimed to facilitate access to employment for vulnerable or disadvantaged groups, such as women or youths. 

On the other hand, telework can lead to unclear boundaries between work and personal life, increased work demands, the depersonalization of relationships at work, a lack of clarity in job roles, and adverse effects on individual wellbeing. Several studies highlight that teleworkers experience greater work intensification, frequent work interruptions, long working hours, lack of recovery time, and more demands to work during one’s free time, at high speed and to tight deadlines, inducing stress and diminishing teleworkers’ wellbeing [5,14]. According to border theorists, the achievement of work–life balance is more difficult where the borders between home and work are intentionally blurred, as is the case for teleworkers [23]. This requirement of constant availability and instantaneous responsiveness, which characterizes many digital jobs, is expected to harm women more than men, as women are those who usually have to juggle work with care, exacerbating inequalities [24,25,26,27].

The expansion of different flexible remote work arrangements in recent years is altering the old profile associated with telework: High-status jobs that enjoy more desirable contracts, afford a high degree of autonomy, are result-oriented, and are in little need of monitoring and control [28]. Teleworking arrangements are now diffusing to more traditional parts of the economy and occupations with a lower status, also expanding among employees with routine tasks that were previously inflexibly tied to the office desk [16,18,29,30]. The diffusion of telework to clerical and low-skilled jobs implies that the working conditions associated with telework should have also deteriorated.

In sum, although an extensive body of research discussing this autonomy paradox associated with telework already exists, potential explanations are still limited [29]. The literature suggests that one way to engage more thoroughly with the autonomy paradox is to distinguish between different types of teleworkers based on the location from where they work [31], yet few studies attempt to test this hypothesis [6,14,17,32]. Therefore, our investigation takes the variability among the various types of remote workers as a main dimension of analysis, as recent research has shown that the level of mobility and the intensity of ICT use varies across telework arrangements, and it has a significant influence on working conditions. Since literature on gendered impacts of telework and digitalization highlight that segregation patterns of the real economy are being replicated in the digital economy, and the different implications for work–life balance of the flexibility associated with remote work [16,33,34,35], we incorporated the interactions between gender and type of telework as a crucial differentiating factor.

Finally, several studies advocate the use of discretion, instead of autonomy, to provide a more nuanced picture of the autonomy paradox and the implications of telework on wellbeing. According to these studies, the possibilities for the individual to choose where, when, and how to work should be more appropriately defined in terms of discretion rather than of autonomy [14,36]. Therefore, we included in our study composite indices of job quality, which allow us to distinguish distinct features of the multidimensional aspects of work organization, working conditions, and impacts on work–life balance, stress, and wellbeing. The analysis of the work intensity index can include quantitative demands, pace determinants, and interdependency and emotional demands. The working time quality index includes duration, atypical working times, working time arrangements, and flexibility. The skills and discretion index captures dimensions related to decision-making, worker participation, and complexity of tasks. The prospects index refers to employment status, career prospects, job security, and downsizing, while earnings refers just to one variable, monthly earnings. 

Accordingly, the analysis of these indices and other information collected in the survey allowed us to test the following:

**Hypothesis** **1**. *Job quality indices vary significantly depending on the type of telework.*

**Hypothesis** **2**. *Job quality index results also differ by the interactions between telework arrangement and gender.*

## 3. Materials and Methods

### 3.1. Sample 

The analyses were based upon data from the sixth European Working Conditions Survey (EWCS) carried out in 2015. This survey is a good source for mapping out the incidence, intensity, and working conditions of teleworkers across European countries from a cross-national perspective. We used the sample of EU28 countries (EU27 plus United Kingdom) composed of 35,765 respondents. 

### 3.2. Dependent Variables

The Sixth EWCS included seven job quality indices that cover extrinsic and intrinsic job features captured from an objective perspective. They are based on positive and negative self-reported features of the job, which measure the concrete experiences of work and have been proven to have a causal effect—either positive or negative—on the health and wellbeing of workers [20]. This study focused on those dimensions that may be altered more by workplace. Therefore, we selected four of these composite indices: (i) Work intensity index; (ii) working time quality index; (iii) skills and discretion index; and (iv) prospects index. These job quality indices are measured on a scale from 0 to 100. With the exception of work intensity, a higher index score corresponds to better job quality. We added the analysis of a last dimension, earnings, which is measured through the monthly income of workers. In contrast to the other job quality indices, this index is only based on one indicator. 

The first index, work intensity, includes quantitative demands, time pressure, frequent disruptive interruptions, pace determinants, interdependency, and emotional demands. A higher score for work intensity indicates a less favorable situation for the worker. Second, the working time quality index includes the incidence of long working hours, scope to take a break, atypical working times, working time arrangements, and flexibility. Third, the skills and discretion index measures the skills required in the job through 14 indicators that comprise the following dimensions: Cognitive dimension, decision latitude, worker participation in the organization, and training. Fourth, the prospects index measures the continuity of employment as assessed through a person’s employment status and type of contract, job security, and career prospects. 

### 3.3. Independent and Control Variables

In order to analyze how different types of remote work depending on workspace and frequency are positively or negatively associated with job quality, we used the following categories of remote workers: Regular home-based teleworkers, highly mobile teleworkers, and occasional teleworkers. Although telework is not directly addressed in the Sixth EWCS, this survey does include several questions based on the main place of work and the reported use of ICT, which allowed us to create a proxy indicator that captures the incidence of telework and mobile work in all EU Member States. Adapting the definition of telework and mobile work proposed by Eurofound and ILO [15], we combined work location, level of mobility, and high ICT use to categorize three types of teleworkers and mobile workers [16]: (a) Regular home-based teleworkers are those who use ICT devices at least several times a month to work from home, and at all other locations (except the employer’s premises) less often than several times a month; (b) highly mobile teleworkers are those who work with the help of ICT devices at least several times a week in at least two locations other than the employer’s premises, or work daily in at least one other location; (c) occasional teleworkers are those working primarily at the employer’s premises, but occasionally (less than several times a month) work from home or other locations (less frequently and/or at fewer locations).

Besides the groups of workers and gender as factors, we introduced other variables that previous research has shown as significantly related to job quality, work–life balance, and teleworking [37,38,39,40,41]. We added demographic variables such as age, educational level, living with a partner, and the presence of children under 15. We also included various employment-related characteristics, such as occupational level, knowledge intensive activities (including high-tech industry and knowledge-intensive services), employment status, working part-time, and years of experience. Likewise, to capture the effects of the national social protections and care regimes, the country variable was included, grouped according to the usual typology of welfare and/or care regimes [17,42]. A good public care infrastructure and the existence of accommodating working time arrangements help workers to balance the dual demands of work and family. The definitions of all these variables are found in Table A1, Appendix A.

### 3.4. Methods of Analysis

To test the theoretical hypotheses defined in the previous section, we compare differences in mean values and variances for the indices for each group of teleworkers, gender, and the interactions among the two factors using *t*-tests and univariate and multivariate analysis of variance (ANOVA and MANOVA) tests. We also calculated Ordinary Least Squares (OLS) regressions to determine the relative contribution of telework arrangements together with the other variables potentially related to the indices.

## 4. Results

### 4.1. Differences by Type of Telework

First, we tested the mean values of the four indices and income to check if there were significant differences among the subgroups of teleworkers. The tests show that differences in the mean of the four indices and income are significant between teleworkers and non-teleworkers and among the three types of teleworkers: Home-based teleworkers, occasional teleworkers, and highly mobile teleworkers. The ANOVA tests for each index and type of teleworker as the independent variable confirm the results showing that regular home-based teleworkers, highly mobile teleworkers, occasional teleworkers, and traditional workers statistically differ for the five indices. Results are for the intensity index (F test = 254, *p* < 0.000), working time quality index (F test = 197.99, *p* < 0.000), skills and discretion (F test = 1781.72, *p* < 0.000), prospects (F test = 190.02, *p* < 0.000), and income (F test = 2514.70, *p* < 0.000). Tukey’s post hoc procedure also reveals that all the groups of workers are statistically different from each other for the five indices at a level at least equal to 5%, except home-based versus occasional teleworkers in working time quality, skills and discretion, and prospects indices. In the prospects and earnings indices, home-based versus highly mobile teleworkers was also not statistically different. The MANOVA test, which simultaneously considers all the composite indices, yielded to the same conclusion (F = 377.43, *p* = 0.000, Wilks’ lambda = 0.8307).

**Figure 1:**
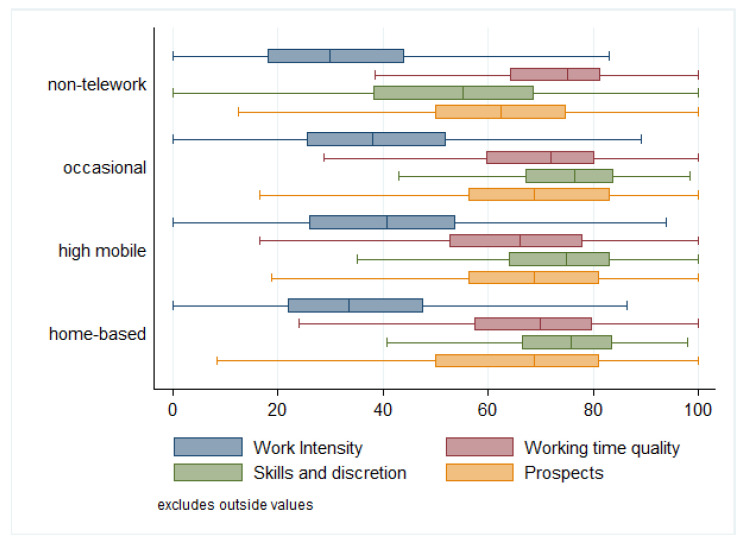
Distribution of indices by type of telework in the EU28 (weighted). Note: The boxes show the range of the values of the index for the middle 50% of the respondents. Lines show the range P5 to P95 for the middle of 90% of the respondents. Source: Own elaboration based on Sixth European Working Conditions Survey (EWCS) data.

As shown in Figure 1, highly mobile teleworkers present the highest value for work intensity (41.4), followed by the occasional teleworkers (39.8) and home-based teleworkers (35.7). Highly mobile workers report greater quantitative demands, particularly regarding working to tight deadlines: 51.6% of highly mobile workers, in contrast to 34% of traditional workers. For the working time quality index, highly mobile teleworkers are the workers with the worst mean value (64.3). Indeed, 23% of them report having a poor work–life balance, compared to only 18% of the rest of the workforce. The second worst group in terms of working time quality is home-based teleworkers (67.4), followed closely by occasional teleworkers. On the contrary, home-based teleworkers present the highest value (74.1) in the index on skills and job discretion, occasional teleworkers are very close, highly mobile teleworkers have the third highest score (71.9), and almost 20 points below are non-teleworkers. Similarly, all teleworkers declare better prospects than traditional workers, occasional teleworkers being those with the highest value (69.8), followed by the highly mobile (68) and home-based teleworkers (66.2). 

### 4.2. Differences by Type of Telework and Gender

Examining gender inequalities within the different groups of teleworkers, *t*-tests resulted in significant disparities by gender in all indices and monthly income, except for the prospects of highly mobile teleworkers and work intensity for highly mobile and home-based teleworkers. Two-way ANOVA tests were also computed for the five indices and the interaction between subgroups of teleworkers and gender. Results showed that there was a significant interaction between the effects of gender and telework arrangement on work intensity (F = 5.97, *p* = 0.0005), skills and discretion (F = 6.63, *p* = 0.0002), prospects (F = 2.78, *p* = 0.0397), and income (F = 15.59, *p* = 0.000). Only the interaction for the working time quality index was not significant (F = 0.52, *p* = 0.6706).

**Figure 2:**
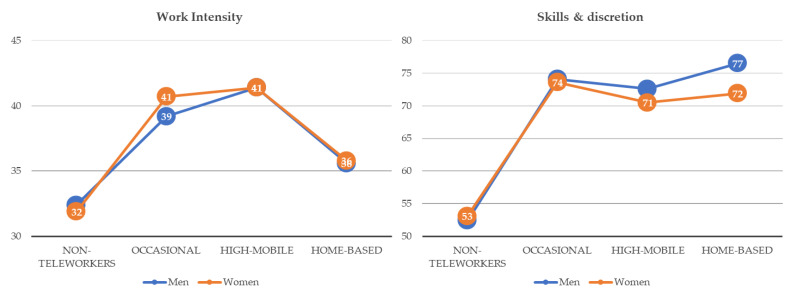

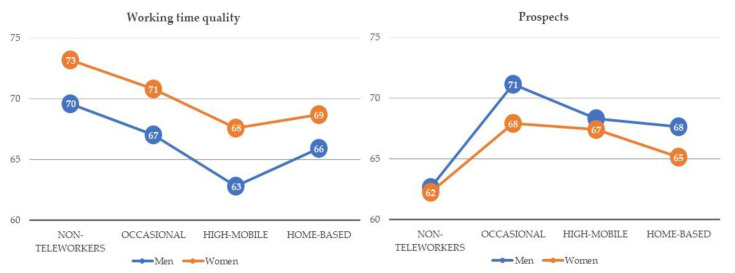
Mean values of the indices by type of telework and gender in the EU28 (weighted). Source: Own elaboration based on Sixth EWCS data.

**Figure 3:**
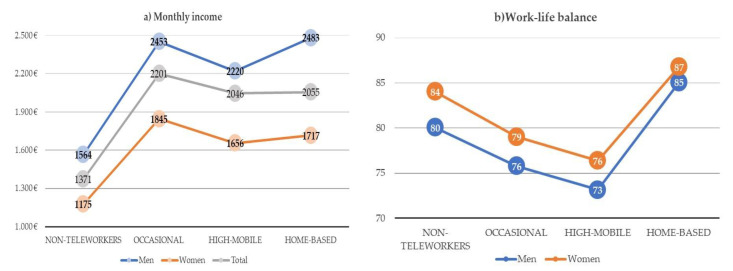
(**a**) Mean values of monthly income (in euro), and (**b**) work–life balance (in % of workers) by type of telework and gender in the EU28 (weighted). Source: Own elaboration based on Sixth EWCS data.

Figure 2 and Figure 3 show the mean scores of the five indices for female and male workers by telework arrangement. Female workers present worse results in all dimensions except for working time quality, but as mentioned, the interaction is not significant. Despite their better scores in this index, women reported lower values when being highly mobile or working at home. There were hardly any gender differences in work intensity index, except in the case of occasional teleworkers, for whom simple main effects analysis showed that the female mean score exceeds that of men (*p* = 0.0000). Regarding skills and discretion, gender gaps were significant and negative for women when home-based teleworking (*p* = 0.001), being highly mobile (*p* = 0.022), and occasional teleworking (*p* = 0.030). Prospects were better for male teleworkers, especially for home-based male teleworkers (*p* = 0.049), while gender gaps in earnings were always significant and negative for women (*p* = 0.000 in the three groups of teleworkers). 

The two-way MANOVA test confirmed these results; specifically, it showed a statistically significant interaction effect between gender and type of telework on the combined indices (F = 5.47, *p* = 0.0000; Wilks’ Λ = 0.9972).

Monthly income is particularly higher for male workers, and gender wage gaps increase for teleworkers, particularly for occasional teleworkers and home-based teleworkers (Figure 3a). The biggest gender gap, in absolute and relative terms, is presented by home-based teleworkers, with women earning 766 euro less each month, or 31% less than men’s wages. Though traditional female workers are those with the lowest salary, with a mean of 1175 euro per month, their gender gap is the lowest in absolute terms (a difference of 389 euros) and the second lowest in relative terms (24.9), just after occasional teleworkers. Figure 3b presents the percentage of workers who report a good fit between their work and their family or social commitments as a proxy for work–life balance, following the Eurofound methodology [20]. Particularly, highly mobile teleworkers report the worst work–life balance, followed by occasional teleworkers. Home-based teleworkers present the best fit between their work and their family or social commitments, both men and women.

To advance in the analysis, we calculated OLS regressions in order to determine the relative contribution of the three types of telework, together with other factors, to the job quality indices. Table 1 shows the optimal specification in terms of explained variance for the two indices with the best results: Skills and discretion (adjusted R^2^ = 0.4194) and prospects (adjusted R^2^ = 0.1531). We report both unstandardized and standardized coefficients of the optimal model to explore the statistical relevance of the estimated coefficients and their relative importance to determining skills, discretion, and prospects. The variance inflation factor (VIF) of each regressor was less than 2.8 in both models, suggesting no multi-collinearity problem in these regressions. We omit some other tests we performed with other variables and the interactions among them, since the model did not improve. 

The results of the regressions reflect the strong effects of teleworking arrangements, which were statistically significant for both indices (with a significance level of 99%). Categories of teleworking have high explicatory effects, showing that job quality indices are different depending on the type of telework. The skills and discretion index is positively associated with teleworking, particularly with highly mobile telework (B = 0.101, *p* ≤ 0.01), followed by occasional (B = 0.087, *p* ≤ 0.01) and home-based telework (B = 0.052, *p* ≤ 0.01). Regarding prospects, highly mobile telework (B = 0.027, *p* ≤ 0.01) and occasional telework (B = 0.025, *p* ≤ 0.01) significantly increase this index. On the contrary, home-based telework (B = −0.017, *p* ≤ 0.1) is negatively associated.

Being a woman reduces the skills and discretion index (B = −0.002, *p* ≤ 0.01) as well as age (B = −0.058, *p* ≤ 0.01) and part-time employment (B = −0.049, *p* ≤ 0.01). On the contrary, self-employment (B = 0.169, *p* ≤ 0.01), secondary education (B = 0.109, *p* ≤ 0.01), and tertiary education (B = 0.188, *p* ≤ 0.01) and years of experience (B = 0.107, *p* ≤ 0.01) show significant positive effects with the skills and discretion index. Occupational categories and economic activities play an important role. By occupation, professionals (B = 0.337, *p* ≤ 0.01), technicians (B = 0.274, *p* ≤ 0.01), and managers (B = 0.226, *p* ≤ 0.01) rank highest. Knowledge-intensive services are positive and statistically significant, except for knowledge-intensive market services, while knowledge intensive industrial activities are not statistically significant, except for low-technology industries, which have a negative relation (B = −0.032, *p* ≤ 0.01).

Regarding the prospects index, being a woman (B = −0.019, *p* ≤ 0.05) and age (B = −0.148, *p* ≤ 0.01) also have a negative effect in the index as well as being self-employed (B = −0.127, *p* ≤ 0.01), part-time employment (B = −0.088, *p* ≤ 0.01), and working in South Europe (B = −0.199, *p* ≤ 0.01) or Eastern countries (B = −0.042, *p* ≤ 0.01). On the contrary, education and experience are positively associated with prospects. Higher occupations present better prospects, particularly professionals (B = 0.108, *p* ≤ 0.01). Knowledge-intensive financial services, market services, and high-technology industries have a positive and statistically significant relation, while low-technology industrial activities have a statistically significant negative relation.

## 5. Discussion

Our analysis confirms Hypothesis 1. Each group of teleworkers presents different working conditions and job quality. Highly mobile teleworkers report the worst job quality and work–life balance due mainly to their high work intensity and low flexibility. Occasional teleworkers are the group with the best job quality, while home-based teleworkers occupy a middle position, with fewer problems of work intensity and working time quality in exchange for lower prospects. 

Our results support the social exchange theory in the sense that all telework arrangements lead to work intensification, though in a different degree in exchange for a higher discretion. Highly mobile workers are the group with the highest work intensity in terms of quantitative demands. The percentage of highly mobile teleworkers that never or rarely have enough time to get the job done is almost double that of home-based teleworkers in that situation, 17% compared to 10%. However, we do not find better working time quality or an improvement in work–life balance for all teleworkers, thus also backing up the presumptions of border theorists. Different types of telework have different impacts on working time quality and work–life balance. While highly mobile remote work is largely incompatible with family responsibilities, home-based teleworking gives workers greater autonomy to organize their working time and improve work–life balance, though not without conflicts, particularly for women and families with children. Looking in detail into the dimensions of the composite indices, we can identify the main sources for differences by gender and for each type of telework. Regarding working time quality, highly mobile teleworkers, who are predominantly men, are those with a higher proportion of long working days (10 hours or more a day) and long working hours (48 hours or more a week). They also work more frequently during weekends and at night. On the other side, the home-based group present the best results, though always worse than non-teleworkers. We also find large deviations by gender in these dimensions: 60.2% of home-based male teleworkers, in contrast to 38.2% of women, work 10 hours or more a day. Large gender differences are also detected regarding control and flexibility of teleworkers’ working schedule: 40.6% of home-based male teleworkers are able to take an hour off during working hours to take care of personal or family matters, compared to 36.6% of women. In this sense, the third index, skills and discretion, also collected items on workers’ capacity to control their work organization. Though home-based teleworkers present the best results, more men than women working from home can choose or change the speed or rate of work (86.7% of men over 80.3% of women). Indeed, more home-based women than men have to devote free time to meet work demands (several times a month), unveiling the existence of conflicts between work and family responsibilities. 

Therefore, our multivariate statistical analysis also validates Hypothesis 2, highlighting the relevance of the interactions between types of telework and gender. Our findings support previous research underscoring the distinct profiles of male and female teleworkers [10,17,26]. Men and women use their opportunities of flexible working in different ways, which leads to different outcomes for wellbeing, work–life balance, and work intensification [35]. Although home-based teleworkers, particularly women, score better in work intensity, some dimensions of working time quality, and work–life balance because of a better alignment of employment with family responsibility [43,44,45,46,47], this is accomplished in exchange for less free time, lower earnings, and worse career prospects due mainly to devoting fewer hours to work. 

Women present lower results in the three items that form the prospects index (career prospects, job security, and downsizing). More men than women agree that their job offers good prospects for the three telework categories, and gender gaps are the highest among home-based teleworkers. Almost one in five women who telework at home perceive insecurity (19%), in contrast to one out of six men (14.6%). Job insecurity is recognized as a significant cause of stress [48], and workers who feel insecure in their jobs are less likely to feel they have a good work–life balance [20]. When prolonged, job insecurity can have damaging effects on people’s career paths and health and wellbeing. Consequently, working at home may not actually improve the quality of women’s working life, but rather reinforce traditional gender roles [26,27,49,50]. 

Finally, differences in job quality are also related to the distinct profiles of teleworkers by occupation and sector, with occasional teleworking being more frequent among managers and professionals, highly mobile work among mid-level occupations, and teleworking from home for managerial occupations and female workers [16,17]. Nevertheless, the OLS regressions confirm that telework arrangements play a role in shaping organizational aspects of work regardless of the specific job or occupation under consideration. All these nuanced results give a more complex interpretation of our hypotheses and demand further investigation into the complexities of job quality, the heterogeneity of teleworkers by main work location, and the significant gender differences in terms of employment status, work and care experiences, and work–family balance. 

## 6. Conclusions

Telework has expanded in recent years thanks to digitalization, increasing flexibility within the labor market, and ICT. This trend has intensified exponentially in the last year with the COVID-19 pandemic, which has forced many companies and organizations to offer home-based telework arrangements to most of their personnel. This study contributes to the debate on the micro-level consequences of these novel flexible arrangements of work, providing a quantitative, more nuanced understanding of the implications of telework on different dimensions of job quality and work–life balance. 

The distinction in our research of different dimensions of job quality and several types of telework allows us to make three main contributions. First, teleworkers have higher discretion over their work and better prospects and income at the cost of work intensification and lower working time quality.

Second, the location, level of mobility, and the intensity of ICT use of the different telework arrangements have a significant influence on working conditions. Occasional teleworkers are the subgroup with the best job quality, while highly mobile teleworkers are those with the worst job quality and work–life balance. Home-based teleworkers occupy a middle position. Home-based teleworkers, particularly women, show better results in terms of work time quality and work intensity, but worse results regarding discretion and economic and career prospects. 

This takes us to our third finding: Interactions between gender and type of arrangement are crucial. Profiles of remote workers are very distinctive by gender. Discrimination and segregation in the labor market and the sexual division of work and care still result in different levels of discretion and autonomy and a distinct use of flexibility in working time, leading to a ”forced” improvement of work–family balance for women at the cost of lower incomes and prospects. Therefore, working at home does not actually improve the quality of women’s working life but boosts traditional gender roles. This should be paid attention to in future studies.

These results call for the adoption of specific legislation and regulatory frameworks that address the protection and quality of working conditions for remote workers, taking into account the distinctions by type of telework arrangement and other crucial axis of inequalities, such as gender.

## Figures and Tables

**Table 1:** Ordinary Least Squares regression models.

Regressors	Skills and Discretion Index		Prospects Index	
	Unstandardized Coefficients	Standardized Coefficients	Unstandardized Coefficients	Standardized Coefficients
**Type of telework**				
Occasional	7.923 *** (0.555)	0.087	2.040 *** (0.720)	0.025
Highly mobile	8.457 *** (0.517)	0.101	2.018 *** (0.661)	0.027
Home-based	5.883 *** (0.606)	0.052	−1.750 * (0.936)	−0.017
**Women**	−1.165 *** (0.309)	−0.002	−0.717 ** (0.335)	−0.019
**Age**	−0.099 *** (0.014)	−0.058	−0.228 *** (0.015)	−0.148
**With partner**	1.647 *** (0.326)	0.036	2.426 *** (0.349)	0.059
**Children < 15**	0.804 *** (0.151)	0.039	0.323 ** (0.161)	0.017
**Level of education**				
Medium	4.591 *** (0.448)	0.109	2.181 *** (0.468)	0.057
High	8.495 *** (0.532)	0.188	2.795 *** (0.589)	0.068
**Self-employed**	1.000 *** (0.387)	0.169	−6.832 *** (0.499)	−0.127
**Part-time job**	−2.497 *** (0.391)	−0.049	−4.093 *** (0.425)	−0.088
**Experience**	1.815 *** (0.139)	0.107	2.480 *** (0.151)	0.160
**Occupation**				
Managers	20.806 *** (0.552)	0.226	6.992 *** (0.806)	0.083
Professionals	18.095 *** (0.510)	0.337	5.286 *** (0.599)	0.108
Technicians & assoc. professionals	16.289 *** (0.486)	0.274	4.777 *** (0.521)	0.088
Clerical support workers	7.928 *** (0.559)	0.114	3.840 *** (0.562)	0.061
**Knowledge-intensive services**				
High-tech	2.264 ** (0.956)	0.017	−0.689 (1.023)	−0.006
Market services	−0.083 (0.617)	−0.001	2.005 *** (0.746)	0.025
Financial services	3.586 *** (0.801)	0.029	3.741 *** (0.936)	0.033
Others	1.781 *** (0.399)	0.038	0.057 (0.437)	0.001
**Knowledge-intensive industrial activities**				
High-technology	0.108 (1.641)	0.000	3.451 * (1.826)	0.016
Medium-high-technology	−1.051 (0.917)	−0.009	0.550 (0.888)	0.005
Medium-low-technology	−0.530 (0.795)	−0.005	0.625 (0.815)	0.006
Low-technology	−2.688 *** (0.629)	−0.032	1.478 ** (0.603)	0.019
**Regime**				
Liberal	4.128 *** (0.510)	0.070	2.740 *** (0.582)	0.051
Mediterranean	−2.967 *** (0.386)	−0.059	−9.129 *** (0.419)	−0.199
Nordic	5.132 *** (0.379)	0.051	2.074 *** (0.471)	0.023
Eastern	−1.686 *** (0.376)	−0.032	−2.034 *** (0.387)	−0.042
Adjusted R^2^	0.4194	0.4194	0.1531	0.1531
F-test (pvalue)	0.000	0.000	0.000	0.000

Notes: *** *p* ≤ 0.01; ** *p* ≤ 0.05; * *p* ≤ 0.1. Robust standard errors in parenthesis. Source: Own elaboration based on Sixth EWCS data, EU28.

## Data Availability

All microdata analyzed are from the European Working Conditions Survey, carried out by Eurofound. The Eurofound datasets are stored with the UK Data Service (UKDS) in Essex, UK, and are promoted online via their website.

## Appendix A

**Table A1:** Definitions of independent variables.

Variable	Description
Type of telework	1. Occasional teleworkers; 2. Highly mobile teleworkers; 3. Regular home-based teleworkers and reference value if non-teleworking
Women	Dummy that takes the value 1 for women and 0 otherwise
Age	Age declared by respondents
Level of education	Highest level of education or training successfully completed: 1. Low education: ISCED 0–2; 2. Medium education: ISCED 3–4; 3: High education: ISCED 5–8
With partner	Dummy that takes the value 1 for individuals who live as part of a couple and 0 otherwise
Children <15	Dummy that takes the value 1 for worker who live with children <15 and 0 otherwise
Employment status	Variable that takes value 1 if the respondent is an employee and 2 if the respondent is self-employed
Part-time job	Dummy that takes the value 1 for individuals who work part-time and 0 otherwise
Experience	Years of experience in the current job
ISCO	1. Managers; 2. Professionals; 3. Technicians and associate professionals; 4. Clerical support workers; reference value if they have other occupations
KIA	Aggregation of knowledge-intensive industrial activities: 1. High-technology: National Economic Activity Classification (NACE) 21 and 22; 2. Medium-high-technology: NACE 20, 27–30; 3. Medium-low-technology: NACE 19, 22–25 and 33; 4. Low-technology: NACE 10–18, 31 and 32; reference value if they work in other activities
KIS	Aggregation of high-tech knowledge-intensive services: 1. High-tech knowledge-intensive services: NACE 59–63 and 72; 2. Knowledge-intensive market services (excluding financial intermediation and high-tech services): NACE 50, 51, 69–74, 78 and 80; 3. Knowledge-intensive financial services: NACE 64–66; 4. Other knowledge-intensive services: NACE 58, 75, 84–88, 90–93; reference value if they work in other activities
Care regime	1. Central European countries (ref); 2. Liberal countries; 3. Mediterranean countries; 4. Nordic or Scandinavian countries; 5. Eastern countries
